# The movement of small insects in the convective boundary layer: linking patterns to processes

**DOI:** 10.1038/s41598-017-04503-0

**Published:** 2017-07-14

**Authors:** Charlotte E. Wainwright, Phillip M. Stepanian, Don R. Reynolds, Andy M. Reynolds

**Affiliations:** 1 Rothamsted Research, Harpenden, Hertfordshire, AL5 2JQ UK; 2 Natural Resources Institute, University of Greenwich, Chatham, Kent, ME4 4TB UK

## Abstract

In fine warm weather, the daytime convective atmosphere over land areas is full of small migrant insects, among them serious pests (e.g. some species of aphid), but also many beneficial species (e.g. natural enemies of pests). For many years intensive aerial trapping studies were the only way of determining the density profiles of these small insects, and for taxon-specific studies trapping is still necessary. However, if we wish to determine generic behavioural responses to air movements shown by small day-migrating insects *as a whole*, the combination of millimetre-wavelength ‘cloud radars’ and Doppler lidar now provides virtually ideal instrumentation. Here we examine the net vertical velocities of > 1 million insect targets, relative to the vertical motion of the air in which they are flying, as a succession of fair-weather convective cells pass over the recording site in Oklahoma, USA. The resulting velocity measurements are interpreted in terms of the flight behaviours of small insects. These behaviours are accounted for by a newly-developed Lagrangian stochastic model of weakly-flying insect movements in the convective boundary layer; a model which is consistent with classic characterisations of small insect aerial density profiles. We thereby link patterns to processes.

## Introduction

The predominant mode of migration of small insects, over all but the shortest distances, is via atmospheric transport and, considering the relative abundance of insects, this is arguably the commonest means of migration among all free-living animals. The first systematic characterizations of the distribution of small insects in the atmosphere were made by C. G Johnson and his collaborators, in the late 1940s and 1950s^1^, based primarily on *simultaneous* sampling of aphids at a range of heights over southern England, but also utilizing data from other pioneering aerial trapping studies (e.g. refs 2–4). In fine weather, and above a zone close (~10 m) to the ground where discontinuities occur (see ref. 5.), the vertical profile of insect density was found to decline steadily with height according to a power law, so that there was a significant linear regression of log density on log height; furthermore, the regression coefficient (*b*) was found to be inversely correlated with the temperature lapse rate, i.e., the rate at which temperature changes with height^1, 6^. The exact value of the regression coefficient (*b*) was found to vary between taxa^5^, indicating species- or group-specific flight behavioural capabilities and constraints. The inverse correlation between *b* and the temperature lapse rate means that in an unstable, convective atmosphere with a high degree of upward transport, there will be a *small* negative value of *b* denoting that a larger proportion of the aerial population is to be found high in the air. Conversely, in stable conditions, a large negative value of *b* indicates that the aerial population is tending to accumulate at lower altitudes. As expected, changes in the lapse rate and the associated gradient of aphid density on height occurred during the course of a day, and there was also a seasonal effect with a sharper decrease in aphid numbers with height as summer turned to autumn and the atmosphere became more stable (p. 337 in ref. 1).

The number of aphids taking to the air is dependent, of course, on the numbers of flight-ready individuals being produced from ground sources, and on the suitability of ambient environmental factors (particularly illumination levels, temperature, and wind-speed) for take-off. After their first upward surge of flight takes them through their ‘flight boundary layer’^5^, aphids will be conveyed to the upper air and brought back again to near the ground (where they may alight) very largely by convective circulation in the atmosphere. From his studies, Johnson [p. 348 in ref. 1] estimated that aphids might take an average of ~1–3 h to complete a circuit, while frit flies (*Oscinella frit*) might average about 1 h. Very many small insect taxa are probably similarly adapted to daytime flight in convective conditions, using upward and horizontal air currents to disperse away from their natal sites. Apart from aphids, other taxa predominant in the atmosphere include certain families of small Diptera (e.g. Chloropidae, Drosophilidae, Sciaridae) and Coleoptera (e.g. Staphylinidae), small parasitic Hymenoptera, Thysanoptera and Psocoptera (e.g. refs 1–4, 7).

While the general paradigm of Johnson and colleagues on the daytime windborne migration in small insects is not in doubt, the precise flight behaviour of the migrants at various times during the daily evolution of the convective boundary layer (CBL) needs some clarification. In particular, we need to understand the migrants’ reactions to vertical air currents, and the degree to which they exert enough self-supporting lift as they find themselves successively in convective up- and downdrafts^8–11^. The advent of high-sensitivity millimetric radars, developed to study the properties of clouds has, in combination with very high-resolution wind-profiling, allowed accurate measurements of the velocities of insects in relation to the air-parcel in which they are flying (c.f. refs 11–16) – a feat considered impossible by the early aeroecologists.

Geerts and Miao^12, 13^ used an aircraft-mounted W-band (3-mm wavelength) profiling radar with both nadir and zenith-pointing beams (with the nearest range-gates about ~100 m above and below the aircraft) to measure the vertical motions of clear-air echoes largely due to small insects in the CBL; these were compared with vertical air motions measured with a gust probe on the aircraft. Some rather complex corrections were required to remove unwanted velocity contributions due to the aircraft motion itself. The study demonstrated that small insects resisted ascent in plumes of rising air; this is not surprising in itself, but the finding that this opposition increased *in proportion* to the updraft strength is more unexpected, and begs the question as to how the insects accurately sensed the vertical air currents. Wood *et al*.^11^ used insect observations made with ground-based 35 and 94 GHz cloud radars combined with data from a 905-nm lidar which determined maximum aerosol height, and thus depth of the CBL, for 12 cloudless occasions in the UK, although only one of these occasions was presented in detail. The main finding of ref. 11 was that insects were often visible on the cloud radar at heights above the lidar-indicated maximum height of aerosol particles, indicating that the insects were engaging in behaviour that is inconsistent with the view that they act as passive tracers in the CBL.

In the present study, we use co-located zenith-pointing Doppler lidar and Ka-band (8.6 mm wavelength) dual-polarized profiling cloud radar at a site in Lamont, Oklahoma, USA, during the months of July and August 2015. The combination of instruments provides unrivalled height- and time-resolved measurements of the vertical component of air velocity simultaneously with quantification of the movements of small insects. The chosen dataset is temporally extensive compared to some other millimetre-wavelength radar studies^11–13^ and free from complications due to disturbed weather or low air temperatures, which restrict observations in other places (e.g. the UK^11^), and thus provides a definitive analysis by virtually ideal instrumentation. In addition, we present a Lagrangian stochastic model of dispersion in convective boundary layers which contains a term accounting for the flight behaviours of aphid-type insects consistent with those emerging from our observational radar/lidar findings. The validated model represents a new way of linking insect density profiles in the CBL to individual flight behaviours, and thereby provides a modern context to Johnson’s classic studies. Understanding how flight behaviour contributes to observed density profiles could, for instance, be used to relate numbers of airborne aphids caught by traps on different days, under different atmospheric conditions, to aphid densities available to colonise crops at local and regional scales.

## Methods and observational Results

**Figure 1:**
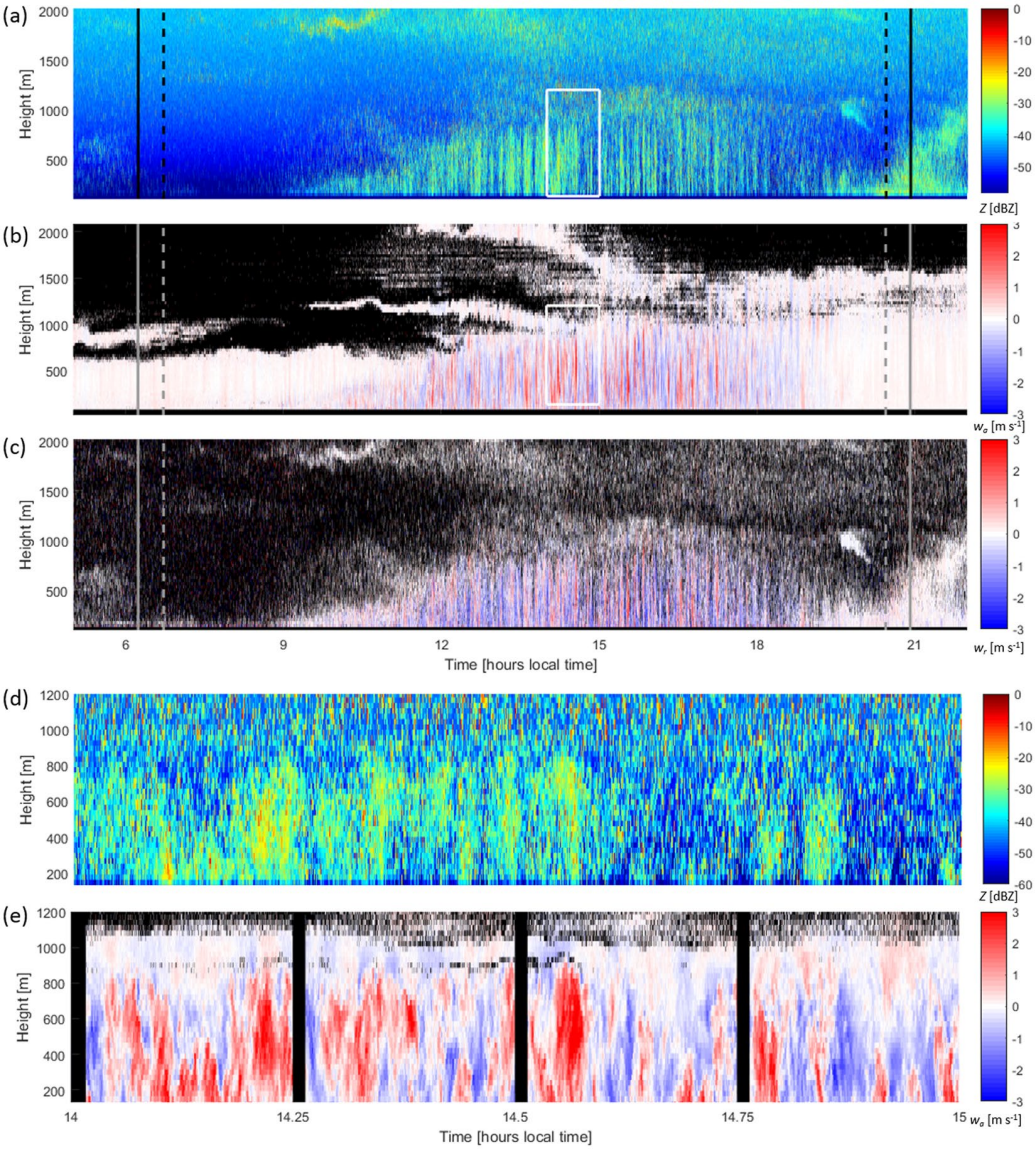
An example case from 12 August 2015. Panel (**a**) shows a time-height profile of reflectivity from the radar, while (**b**) presents the atmospheric vertical motion, *w*_*a*_, measured by the Doppler lidar. Panel (**c**) shows the vertical motion recorded by the radar, *w*_*r*_. For panels (**b**) and (**c**) blue colours represent downward motion and red upward motion. The dashed vertical lines in panels (**a**)–(**c**) indicate local sunrise and sunset while the solid vertical lines show the cessation and onset of civil twilight. The lower two panels (**d**) and (**e**) present a magnified view of the reflectivity and vertical air motion fields outlined by the white boxes in (**a**) and (**b**).

To derive the vertical motion of insects with respect to the up- or downdraft in which they are embedded, we expand on the technique proposed by Geerts and Miao^12, 13^, which compares measurements of atmospheric motion that contain contributions from insect motion to atmospheric measurements unaffected by insects as targets. The Ka-band zenith-pointing radar operated by the Atmospheric Radiation Measurement program in Lamont, Oklahoma, USA provides vertical motion of the insects (Fig. 1c) with time and height resolution of 2.7 s and 30 m respectively. The vertical motion from this radar, *w*_*r*_, is the sum of the true atmospheric motion, *w*_*a*_, and the insects’ own motion, *w*_*i*_. Furthermore, the radar takes polarimetric measurements at two orthogonal linear polarizations using the so-called linear depolarization ratio (LDR) mode. In this configuration, a single linear polarization is transmitted and its copolar and cross-polar backscattered signals are recorded separately, providing the degree of depolarization by the scattering object. Generally, meteorological scatterers produce low depolarization ratios, while biological scatterers produce larger values^17^. For example, for a day containing several periods of precipitation (8^th^ August 2015) the median LDR during the precipitation was −22 dB, with the 5^th^ and 95^th^ percentiles of the distribution at −21 dB and −22.7 dB, while the median LDR for biological echoes was −10 dB with the 5^th^ and 95^th^ percentiles of the distribution at −2.1 dB and −21.4 dB. We compare the vertical motion from Ka-band radar to that from the co-located Halo Streamline Doppler lidar (Fig. 1b), which provides atmospheric vertical motion, unadulterated by insect movements, at 3-s and 26-m resolution. The lowest height that both instruments can reliably see during the daytime is approximately 130 m above the ground. The ‘roughness sublayer’ of the atmosphere at this site will be shallow as it is flat with low vegetation (grasses).

In addition to measuring vertical motion, the Ka-band radar also provides vertical profiles of reflectivity, which in the absence of precipitation can be interpreted as a proxy for animal density in the airspace. An example reflectivity time-height plot in Fig. 1a illustrates typical diurnal boundary-layer growth following sunrise at around 07:00 local time. By comparing Fig. 1d and e, which present magnified views of the reflectivity and vertical air motion in the fully-developed CBL between 14:00–15:00, we see that the plumes of increased reflectivity that form from around 11:00 onwards in the daytime CBL represent insects caught within updrafts, as also observed by Geerts and Miao^12, 13^.

The data used herein were recorded during July and August 2015 between the hours of 14:00 and 18:00 local time (19:00 and 23:00 UTC), to restrict analysis to a fully-developed CBL. As can be seen in Fig. 1, during the period included in our analysis (14:00–18:00 local time) the CBL reaches a depth of approximately 1 km. To restrict the data to periods in which single insects were likely to be present within the radar beam we discarded data with signal-to-noise ratio below 0 or spectral width above 0.1 m s^−1^. Periods of precipitation were removed via a linear depolarization ratio filter, following Martner and Moran^17^ and using a threshold of −15 dB. In total, 1,062,377 individual points across 52 days remained after filtering, each representing a single insect. The Doppler lidar data were interpolated in time and height to match the Ka-band radar data exactly. Subtracting the vertical wind contribution (Fig. 1c) from the overall vertical motion of the insect (Fig. 1b) yields the insect flight behaviour with respect to the background air currents.

**Figure 2:**
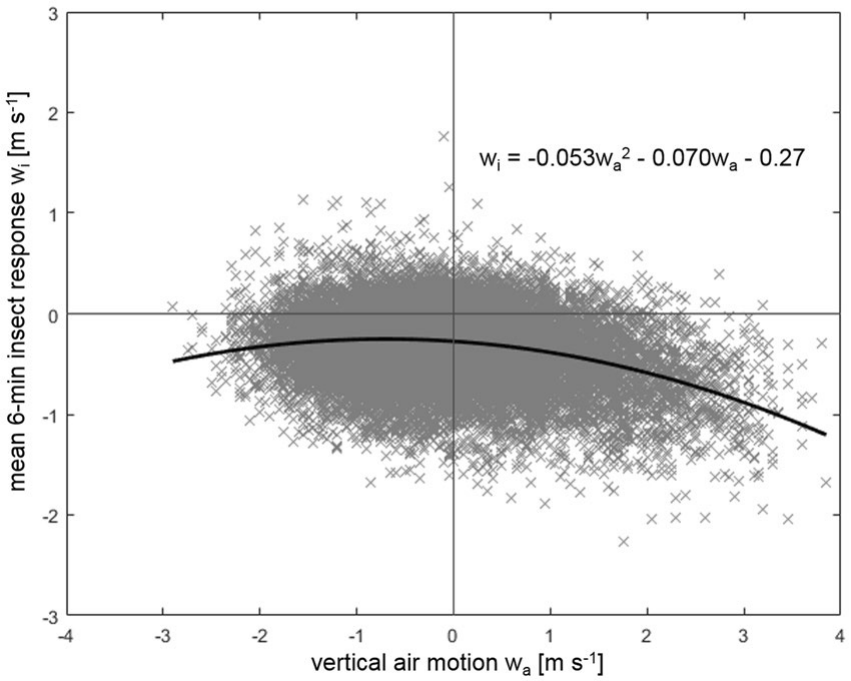
Observed difference between the vertical velocities of small insects and the surrounding airstream in the fully-developed convective boundary layer, based on 29,343 data points. The solid black line indicates the quadratic best fit to the data. The fit was performed using a quadratic linear regression. The parameter standard errors and *p*-values are 0.0022 (*p* = 0) for the *w*_*a*_^2^ term, 0.0022 (*p* ≪ 10^−4^) for the *w*_*a*_ term and 0.0016 (*p* ≪ 10^−4^) for the intercept. The root mean squared error corresponding to the fit is 0.31 m s^−1^ and the *R* ^2^ value is 0.086.

Due to the large number of resulting data points, we follow the example of Geerts and Miao^12^ and separate the insect response (i.e., vertical movement with respect to the vertical movement of surrounding air flow), *w*_*i*_, into bins based on atmospheric vertical velocity, *w*_*a*_. We examine the data at 6-minute intervals due to the fast turnover time of eddies within the CBL. Within each 6-minute interval the data are then separated into 0.05 m s^−1^ bins, with no separation based on height. For each 0.05 m s^−1^ *w*_*a*_ bin containing five or more data points, all *w*_*i*_ measurements falling into that bin are averaged to give a mean *w*_*i*_ value for each time and velocity interval. Not every *w*_*a*_ bin will contain data during every 6-minute interval, and this is particularly true for strongly positive or negative *w*_*a*_ bins (i.e., strong updrafts or downdrafts), which occur with less frequency. Processing in this manner resulted in 29,343 average insect response values. By comparing *w*_*i*_ to *w*_*a*_, we can elucidate the response of the insects to atmospheric vertical motion. In downdrafts (i.e., *w*_*a*_ < 0) the insects are seen to descend with respect to the airstream in which they are embedded at an average rate of 0.25 m s^−1^ (standard deviation 0.30 m s^−1^), with relatively little velocity-dependence (Fig. 2).

In contrast, although insects within updrafts are also descending with respect to the surrounding motion, they do so at a velocity-dependent rate, as indicated by the slope of the best fit line in Fig. 2 for *w*_*a*_ > 0. This effect results in a net average descent rate with respect to the surrounding air of 0.42 m s^−1^ (standard deviation 0.35 m s^−1^) within updrafts. The self-powered descent of the insects is insufficient to mitigate the rising motion in the updraft, but it slows their ascent with respect to the ground. Although this technique allows us to examine the net average behaviour of the small insects with respect to atmospheric vertical motion, the relatively high level of scatter seen in Fig. 2 could indicate the presence of several different species groups with varying masses and flight capabilities within the radar measurements.

### Data Access

The datasets analysed in the current study are available in the DOE ARM Climate Research Facility repository at https://www.arm.gov/capabilities/instruments.

### Theory

Franzese *et al*.^18^ formulated a simple “Lagrangian stochastic” (LS) particle tracking model of vertical dispersion in the convective boundary layer that is in close agreement with laboratory data from carefully controlled experiments. This model simulates the trajectories of individual tracer (flow-following) particles in the turbulent flow. Here we show how this model can be adapted to simulate the trajectories of small insects (referring to insects with mass of approximately 0.1–5 mg). Franzese *et al*.^18^ considered the one-dimensional case of vertical dispersal in stationary and horizontally homogeneous high-Reynolds-number turbulence with no mean flow. Under these conditions, the motions of independent tracer particles in the vertical direction (*z*) can be described by the stochastic differential equations^19^:

1 $$d{w}_{a}=a({w}_{a},z)dt+{[{C}_{0}e(z)]}^{1/2}dW$$

$$dz={w}_{a}dt$$

where *w*_*a*_ is the vertical velocity of the tracer particle, *C*_0_ is a universal constant, *ε*(*z*) is the average rate of dissipation of turbulent kinetic energy, and *dW* are the increments of a Wiener process with zero mean and variance *dt*. The deterministic acceleration term *a*(*w*_*a*_, *z*) is derived from the Fokker-Planck equation^19^

2 $${w}_{a}\frac{\partial g({w}_{a},z)}{\partial z}=-\frac{\partial }{\partial {w}_{a}}[a({w}_{a},z)g({w}_{a},z)]+\frac{{C}_{0}\varepsilon }{2}\frac{{\partial }^{2}g({w}_{a},z)}{\partial {w}_{a}^{2}}$$

where *g*(*w*_*a*_, *z*) = *ρ*(*z*)*P*_*E*_(*w*_*a*_, *z*) is the density-weighted Eulerian probability density function, *ρ*(*z*) is the air density and *P*_*E*_(*w*_*a*_, *z*) is the Eulerian probability density function of the vertical turbulent velocity, *w*_*a*_, at a given height *z*. This specification of *a*(*w*_*a*_, *z*) ensures that initially well-mixed simulated tracer particles (i.e., with probability densities proportional to *g*(*w*_*a*_, *z*)) will remain so. That is, the acceleration of individual particles within the flow (i.e., modelled Lagrangian motions) are defined to maintain height distributions prescribed by the Eularian input definitions of the boundary layer velocity statistics. This condition is equivalent to or more stringent than all other conditions (physical constraints), identified thus far, that should be satisfied by LS models^19^. Franzese *et al*.^18^ assumed a simple parameterization for the deterministic acceleration term

3 $$a({w}_{a},z)=\alpha (z){{w}_{a}}^{2}+\beta (z){w}_{a}+\gamma (z)$$

where the parameters, *α*(*z*), *β*(*z*), and *γ*(*z*) are determined by a system of equations obtained by multiplying the Fokker-Planck Equation, Eq. (2), successively by powers of *w*_*a*_, and then integrating over velocity:^18^

4 $$\alpha (z)\,\bar{{w}_{a}^{n+1}}+\beta (z)\,\overline{{w}_{a}^{n}}+\gamma (z)\,\overline{{w}_{a}^{n-1}}=\frac{1}{n}\frac{\partial \overline{{w}_{a}^{n+1}}}{\partial z}+\frac{1}{n}\frac{\overline{{w}_{a}^{n+1}}}{\rho }\frac{\partial \rho }{\partial z}+\frac{n-1}{2}{C}_{0}\varepsilon (z)\overline{{w}_{a}^{n-2}}\quad n=1,2,3$$

The second term on the right-hand side of Equation (4) does not feature explicitly in Franzese *et al*.^18^ because they implicitly assumed that away from sources the tracer-particle density is constant within the convective boundary layer. The deterministic acceleration is thus completely defined by the first four Eulerian moments of velocity, $\overline{{w}_{a}^{n}}$, which are known from experiment. Franzese *et al*.^18^ provided parameterizations for these models. The LS model reduces to the standard exact form when turbulent velocities are Gaussian, and works well under moderate skewness conditions observed in the convective boundary layer^18^. A key advantage of this modelling approach is that, unlike other LS models for the convective boundary layer (e.g. ref. 20), the use of turbulence statistics up to the fourth order can be made without assuming any predefined form for the probability distribution function for the velocity. The functional forms of these other models are sensitively dependent upon the mathematical form of this probability distribution function and can behave erratically when velocities are high^21^.

We modified the model of Franzese *et al*.^18^ to make it more appropriate for the vertical dispersal of small insects in the convective boundary layer. We did this by assuming that the key distinction between small insects and tracer particles lies with their respective density profiles. When passive tracer particles are distributed non-uniformly though the depth of a convective layer, the effect of turbulent atmospheric motions is to diffuse particles into a homogeneous height distribution. The result of turbulent mixing is that tracer-particle densities remain nearly constant away from sources. Unlike passive tracers, numbers of small insects are observed to decrease with altitude (see Introduction) as they actively resist the diffusing actions of turbulent mixing. We assume further that the moments of the velocity distributions characterizing the vertical motion of small insects embedded within the flow (i.e., *w*_*r*_) can be modelled using the same formulation as that of tracer particles, permitting the adoption of a LS model following that of Franzese *et al*.^18^ With these assumptions, the deterministic acceleration coefficients of tracer particles, *α*(*z*), *β*(*z*) and *γ*(*z*), are replaced by modified acceleration coefficients *α*(*z*) + *α’*(*z*), *β*(*z*) + *β’*(*z*), and *γ*(*z*) + *γ’*(*z*) where the new, additional density-dependent terms are obtained by solving Equation 4:

5 $$\alpha ^{\prime} =[-\frac{{\overline{{w}_{a}^{3}}}^{2}}{2{\overline{{w}_{a}}}^{2}}-{\overline{{w}_{a}^{2}}}^{2}+\frac{\overline{{w}_{a}^{4}}}{3}]/[\overline{{w}_{a}^{4}}-\frac{{\overline{{w}_{a}^{3}}}^{2}}{\overline{{w}_{a}^{2}}}-{\overline{{w}_{a}^{2}}}^{2}]\frac{d\,\mathrm{ln}\,\rho }{dz}$$

$$\beta ^{\prime} =\frac{\overline{{w}_{a}^{3}}}{2\overline{{w}_{a}^{2}}}\frac{d\,\mathrm{ln}\,\rho }{dz}$$

$$\gamma ^{\prime} =\overline{{w}_{a}^{2}}\frac{d\,\mathrm{ln}\,\rho }{dz}$$

and represent the response of insects to atmospheric motion and their local aerial concentrations. These additions stem from the second term on the right-hand side of Equation 4, the term that Franzese *et al*.^18^ omitted. In (5) *ρ*(*z*) now denotes the aerial density of the insects which can be characterised by power laws with taxa or species-specific regression coefficients^1, 10^.

The accelerations of small insects and the surrounding air are therefore predicted to differ by an amount

6 $$a\mbox{'}({w}_{a},z)=\alpha \mbox{'}(z){{w}_{a}}^{2}+\beta \mbox{'}(z){w}_{a}+\gamma \mbox{'}(z)$$

**Figure 3:**
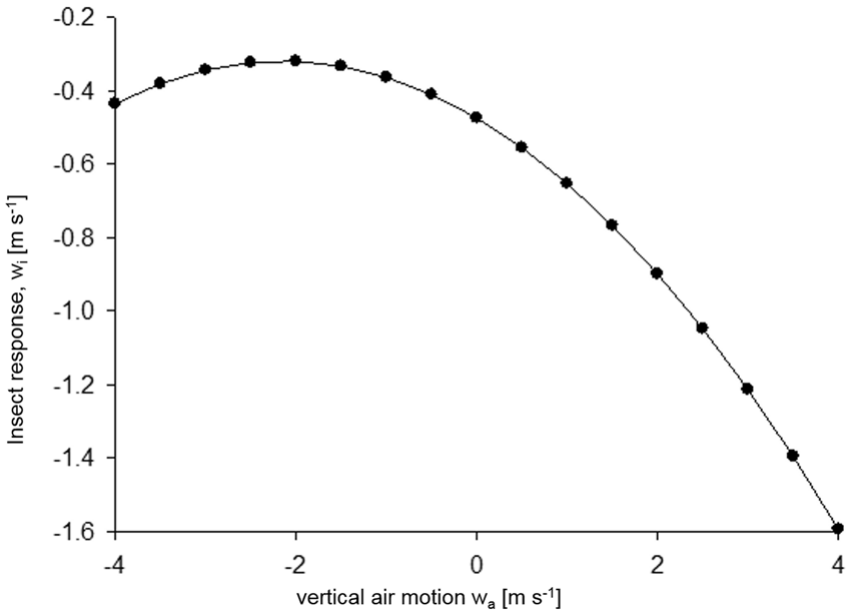
Predicted difference between the vertical velocities of aphid-size (~0.5 mg) insects and the surrounding airstream in the middle of a convective boundary layer. The magnitude of the response is dependent upon the aerial density profile, the convective velocity scale (surface heat flux), and acceleration autocorrelation timescale. The shape of the response curve is determined by the statistical properties of the turbulence (Eqs 5 and 6) which are common to all convective boundary layers.

This additional acceleration represents a driving downward force (i.e., towards higher aerial densities) that allows for the maintenance of non-uniform aerial density profiles. Without this force, gradients in aerial densities would eventually get smoothed out as there would be nothing to counter the tendency of turbulent dispersal to drive small insects upwards (i.e., towards lower aerial densities). The acceleration term, Equation 6, can be regarded as encapsulating an active response of small insects to the surrounding air flow causing an additional change in velocity, *du*’≡ *w*_*i*_ ≈ *a*’(*w*_*a*_, *z*)*dt*, beyond that caused by following the air flow, where *dt* is the time over which accelerations remain significantly correlated. Model predictions for the dependencies of these velocity differences on the instantaneous air velocities (Fig. 3) are in close accord with our observations (Fig. 2).

For Gaussian turbulence (i.e., for $\overline{{w}_{a}^{3}}=0\,\,$and $\overline{{w}_{a}^{4}}=3{\overline{{w}_{a}^{2}}}^{2}$), the expression in Equation 6 reduces to the exact, velocity-independent, expression that was examined by Stohl and Thomson^22^ albeit in a different context. In those circumstances, which pertain to stable boundary layers, *α’* = *β’* = 0 and as a consequence insect responses are predicted to be velocity-independent. Initial examination of corresponding stable boundary layer data from the two-month period outlined in the previous section showed velocity differences reduced by 80% as compared to those in the CBL.

## Discussion

The seminal field studies led by C. G. Johnson in the 1940s and 1950s led to a simple characterization of aphid aerial density that diminishes with height above the ground such that the linear regression coefficient, *b*, of log density on log height provides a single-parameter characterisation of the vertical density profile^6, 23^. This empirical relationship was later found to fit density profiles of a variety of other small insects^1, 6^. The key to understanding the biological, ecological and evolutionary consequences of these profiles lies with the elucidation of the underlying generative processes.

Here we reported on new observations of the vertical motion of insects with respect to the up- or downdrafts in convective boundary layers. We found that insects are typically moving downwards through the downdrafts and are typically moving upwards when in the updrafts albeit at a slower pace than the air itself (Fig. 2). What are the implications of these results for a typical migratory flight of a small, say, aphid-size insect? As expected, insect migrants are taking off throughout the convective period of the day, presumably as soon as they become physiologically flight-ready (see p. 279 in ref. 1); there is no concerted period of take-off as can be seen around dusk (Fig. 1) for night-flying taxa. Small day-flying insects will be taking off from the plant canopy with a range of climb rates – for example, *Aphis fabae* still flying after 10 minutes ascended at about 0.14–0.22 m s^−1^ ^24^. At anything like these speeds, the insect will quickly reach altitudes (~10 m from the canopy) affected by turbulent convection. If it encounters an updraft (of perhaps, ~1 m s^−1^) it will, perforce, be borne upwards. We infer that for many individuals, self-propelled ascent soon changes to a sort of energy-saving ‘hovering’, which (in still air) would not fully counteract gravity. For aphids this probably occurs at a height where there is a balance between attraction to short-wavelength light from the bright sky compared to weaker long-wavelength light from vegetation below^8^. Whatever the mechanism, our results clearly show that, although insects continue to fly, they do not keep pace with the ascending air. Moreover, we know from other studies^1^ that on days when turbulent convection is absent, a vertical distribution characteristic of occasions when cumuliform eddies are present does not occur, and aphids are restricted to within a few tens of metres of the surface. This unenergetic mode of migration is presumably an adaptation to daytime flight in numerous species of small insects; it is not an intrinsic ‘design limitation’ because relatively small *nocturnally-flying* insects such as the brown planthopper (*Nilaparvata lugens*) can ascend to high altitudes under their own power and maintain flight there for many hours^25^.

If carried to altitude by a thermal plume, an insect will eventually encounter an area of descending air; these downdrafts are weaker, but more extensive in area, than the updrafts. We found that insects in general are typically moving downwards through the downdrafts at 0.25 m s^−1^, prompting the question of how this rate compares to measured sinking speeds of small insects. Thomas *et al*.^26^ made careful estimates by releasing freshly anaesthetized aphids (*Aphis fabae* of average weight 0.49 mg) into updrafts of known speed in a laboratory flight chamber. The mean terminal velocity was 1.78 m s^−1^ with wings closed and 0.82 m s^−1^ with wings fully extended horizontally. These measurements are in agreement with two terminal velocity values obtained by timing the drop, in still air, of live *A*. *fabae* with wings closed (1.83 m s^−1^), and dead ones with wings extended (0.92 m s^−1^) mentioned in ref. 1 (p. 355). Terminal velocities in turbulence will be smaller, because such velocities are flow-dependent (e.g. ref. 27). However, it seems clear that the insects we observed were not tumbling downwards with closed wings. They may have been spiralling down, abdomen first, with extended wings in a state of reflex immobility (see; ref. 26; small insects like aphids do not glide), but this means of descent still seems rather fast compared to what we have observed. The most likely possibility is that they were maintaining wing-beating, but with not enough effort to maintain altitude. Alternatively they could have been flying downwards – Thomas *et al*.^26^ found a mean maximum speed of 0.38 m s^−1^ for downward flying *A. fabae* when the floor of the flight chamber was coloured orange (slower speeds were found with a green-coloured floor).

Our results on the vertical movements of insects in up- and downdrafts are predicted by a newly-formulated Lagrangian particle-tracking model which accounts explicitly for the decrease in insect numbers with increasing height as observed by Johnson^6^ and is also consistent with our observations of turbulent dispersal in the convective boundary layer (Fig. 2). We have thereby linked Johnson’s aerial density profiles to flight behaviours; i.e., we have linked observed patterns to behaviour which is a key objective of movement ecology^28^.

Our observations are consistent with those of Geerts and Miao^12, 13^ using data from an airborne Doppler radar. We, like Geerts and Miao^12, 13^, find that the insect response (i.e., vertical movement with respect to the vertical movement of surrounding air flow) increases with the updraft strength (Fig. 2). Our theoretical analysis suggests that these observations and Johnson’s aerial density profiles are two sides of the same coin, as anticipated to some extent by Reynolds and Reynolds^10^.

Geerts and Miao^12^ proposed, somewhat counter-intuitively, that small insects (<10 mg) actively oppose being taken up in convective plumes, and furthermore that this opposition increases with updraft strength. This interpretation of the data is broadly consistent with that of Reynolds and Reynolds^10^ who suggested on the basis of turbulence modelling that aphids produce just enough lift to become neutrally buoyant when they are in updrafts and cease to produce lift when they are in downdrafts. Our new observations indicate that this assertion can now be replaced by the weaker, more nuanced assertion that small insects typically travel upwards within updrafts but do not keep pace with the air flow, slowing their ascent at a rate proportional to the updraft strength. This characteristic need not be attributed to insects actively opposing upward movement as this would be an over-interpretation of the observations. It could, for example, stem from the inability of small insects to fully counter gravity when being buffeted by turbulence within the updrafts – a hindrance to lift generation which can be expected to increase as the vertical air motion strengthens. Lift may also be affected by altitudinal changes in air density, which roughly corresponds to the changes in pressure with height within the CBL since the variation in absolute temperature (~300 K) throughout the CBL is small. This explanation requires that small insects can distinguish between upward and downward movements. This is possible because some insects, including an aphid (*Macrosiphum euphorbiae*), can sense weather changes from air pressure changes^29^; changes which are comparable to the typical air pressure drops with altitude of about 100 hPa over the first 1000 m.

Our observations provide strong support for a new theory of small insect dispersal. Our new theory goes beyond that of Reynolds and Reynolds^10^, who, by comparing aphid density distributions with a Lagrangian Stochastic particle dispersion model surmised that aphids were generating sufficient lift to become neutrally buoyant in updrafts while not generating lift when within downdrafts, but left open the major challenge of migration behaviour for other migratory insects. Taylor^5^, for example, found that the regression coefficients for Homoptera (mainly aphids) were plainly different from other major taxa such as Diptera and Coleoptera measured over the same period, showing that flight behaviours are specific to species or small groups of species. The formulation developed in Equations 1–6 show how these behaviours can be inferred from observations of aerial density profiles and atmospheric dispersion models, although aerial sampling is still necessary for determining taxonomic composition. Within this framework, the airborne dispersal of weak fliers may now be predicted more reliably on the basis of aerial density profiles and atmospheric physics. This holds promise of a new generation of precision pest forecast models driven by high resolution profiling data which should become available with the development of millimetre-wavelength entomological radars.

### Doppler lidar data

Atmospheric Radiation Measurement (ARM) Climate Research Facility archive doi:10.5439/1025185.
